# Knowledge of risk factors, beliefs and practices of female healthcare professionals towards breast cancer in a tertiary institution in Lagos, Nigeria

**DOI:** 10.1186/1471-2407-9-76

**Published:** 2009-03-04

**Authors:** Nasiru A Ibrahim, Olumuyiwa O Odusanya

**Affiliations:** 1 Department of Surgery, Lagos State University College of Medicine and Lagos State University Teaching Hospital, Ikeja, Lagos, Nigeria; 2 Department of Community Health and Primary Health Care, Lagos State University College of Medicine and Lagos State University Teaching Hospital, Ikeja, Lagos, Nigeria

## Abstract

**Background:**

Breast cancer is the leading female malignancy in Nigeria. Screening for early detection has led to reduction in mortality from the disease. It is known that attitudes of physicians and motivation by community nurses influence uptake of screening methods by women. This study aims to investigate knowledge of breast cancer risk factors, beliefs about treatment and practice of screening methods among a cohort of female healthcare professionals in Lagos, Nigeria.

**Methods:**

A cross-sectional study was conducted using a self-administered questionnaire to assess the knowledge of breast cancer risk factors, beliefs about treatment and practice of screening methods among 207 female doctors, nurses and other healthcare professionals working in a university teaching hospital in Lagos, Nigeria. Stratified random sampling method was employed. Chi square test, analysis of variance and Mantel-Haenszel test were performed in data analysis using SPSS v10.0 and Epi Info version 6 statistical packages.

**Results:**

Female doctors obtained a mean knowledge score of 74% and were the only professional group that had satisfactory knowledge of risk factors. Majority (86%) believed that early breast cancer is curable while half of participants believed that prayer can make breast cancer disappear from the affected breast. Eighty three percent practice breast self-examination (BSE) once a month and only 8% have ever had a mammogram. Age, knowledge of risk factors, profession and beliefs were not significantly associated with rate of BSE in this study.

**Conclusion:**

Results from this study suggest the need for continuing medical education programmes aimed at improving knowledge of breast cancer among female healthcare providers other than doctors.

## Background

Breast cancer is the most prevalent cancer worldwide with about 1 million new cases annually [1]. In Nigeria, it has overtaken cancer of the cervix to become the commonest malignancy in women [2]. Many countries are recording increase in incidence rates of breast cancer, with greatest changes in developing countries where rates were initially low [1].

Awareness and knowledge about breast cancer vary among communities and population groups worldwide. While studies conducted to assess breast cancer knowledge among women showed satisfactory level in some places [3,4], other reports, especially from developing countries like Nigeria revealed inadequate knowledge and awareness about the disease [5,6]. Although, patients in communities with high level of awareness usually present with less advanced stages of breast cancer as a result of adoption of screening methods [1], those in communities with low level of awareness often present late [2,7].

Early detection and treatment of breast cancer is associated with better chance of long-term survival [8]. In Nigeria, about two-third of patients with this disease present with advanced stages when therapy offers minimal benefit [2,9]. Reports from Western Europe and North America revealed reduction in mortality from breast cancer due to adoption of screening methods for detection of early diseases [1,10].

Breast self-examination (BSE), clinical breast examination (CBE) and Mammography are recognized screening methods for breast cancer [11]. However, uptake of these methods by women depends on several factors including religious beliefs [12], attitude of their physicians to breast cancer screening and motivation by the community nurses [13]. Female healthcare professionals have greater influence on women's positive perception of breast cancer and motivation to practice screening methods for early detection of the disease [14]. In addition, level of knowledge and attitudes of healthcare providers towards breast cancer are important determinants of their influence on adoption of screening method by women in their localities [13,15]. Reports have shown varying levels of knowledge about breast cancer among healthcare workers [15-17]. Improving their knowledge and screening practices through targeted interventions have positively influenced adoption of early detection methods by women in their communities [13].

In Nigeria, previous studies on breast cancer knowledge assessment were conducted mainly among community dwelling women [5,18]. Reports from these studies showed low level of awareness of breast cancer and practice of screening methods. In view of the large proportion of patients with breast cancer in Nigeria presenting with advanced stages of the disease, there is need for more awareness of measures for early detection. Adequate knowledge and positive attitude towards breast cancer screening are essential for female healthcare professionals if they are to play their expected role in breast cancer awareness campaign in Nigeria. Studies in Nigeria on knowledge, attitude and practice of healthcare providers towards breast cancer are few. In one of such studies, Nigerian nurses were found to be very knowledgeable in several aspects of breast cancer but did not fully understand the risk factors [19]. In a community-based study, two-third among participating female healthcare professionals practised BSE only occasionally and only one doctor could describe how to perform BSE correctly [20]. Therefore, there is need to assess current level of knowledge of breast cancer risk factors and practice of screening methods among female healthcare professionals. This would help in determining the need for continuing medical and health education programmes that could improve knowledge of the disease and adoption of early detection measures among this group of healthcare providers. This will not only enhance their positive influence on women but also improve their individual level of breast awareness. The objective of this study is to assess knowledge of breast cancer risk factors, beliefs about treatment and practice of screening methods among female healthcare professionals.

## Methods

The study was designed as a cross sectional survey of female healthcare professionals. The aims were to assess level of their knowledge about breast cancer risk factors, beliefs concerning breast cancer treatment and their practice of BSE, CBE and Mammography as screening methods.

### Participants

The study was conducted in January, 2007 at Lagos State University Teaching hospital in Lagos, Nigeria. The institution had 652 female healthcare professionals consisting 550 nurses, 64 doctors and 38 allied healthcare providers. Participants were selected using stratified random sampling method with proportional allocation. Sample size was calculated to achieve 95% confidence and 5% type 1 error. A total number of 207 participants were selected among all eligible healthcare professionals in ratio 7:2:1 for nurses, doctors and others respectively. Questionnaires were distributed to participants through their sectional heads using the attendance list in a monthly hospital programme. Names of those who participated were randomly selected from the list until the sample size allocated to each professional group was achieved. All those selected returned their questionnaires within 24 hours to their respective heads of section and were retrieved the same day they were returned. Written informed consent was obtained and assurance of confidentiality of responses was given to each respondent. The hospital Research and Ethics committee approved the conduct of the study.

### Measurements

A self administered questionnaire prepared by the author was employed. Questions were partly drawn using information on breast cancer from the literature. Additional questions were adapted, after modification, from questionnaires used in similar studies conducted earlier in the country. Pre-test was conducted on 15 female healthcare professionals working in the hospital following which some questions were modified to improve clarity. Those that participated in the pre-test were not part of the study. The questionnaire was in four parts. The first part was to elicit socio-demographic data on age, profession, marital status and qualification of each study participant. Questions relating to knowledge of breast cancer risk factors were asked in the second part. Eight items with different levels of relative risk for breast cancer were included and they were to generate "Yes" "No" or "Don't know" response. Participants awareness of breast cancer and early detection methods were also assessed in this section. The third part of the questionnaire assessed participant's beliefs about breast cancer treatment. In the last part, questions were asked on the practice of BSE, CBE and Mammography among participants. Most of the questions in the third and fourth parts were also designed to elicit similar response of "Yes" "No" or "Don't know".

### Analysis

Each respondent was scored on knowledge of risk factors based on the number of correct answers (n = 8). Out of a total score of 100, percentage score for each participant and mean score for different professional groups were calculated. Knowledge score of all the groups were compared while that of the two dominant groups i.e. nurses and doctors were also compared separately. A score of > 80% was considered as indicating "excellent" knowledge, scores between 60% and 80% was labelled "very good" knowledge, scores between 40% and 60% represented "good" knowledge while a score of < 40% was classified as "poor" knowledge. Response of participants belonging to different professional groups to questions on beliefs and practice of screening methods were also compared.

SPSS V10.0 Statistical package was used in data analysis. Frequency distributions were produced for the variables. Statistical comparison was carried out using chi-square test for qualitative variables and proportions and analysis of variance for quantitative variables. Epi Info version 6 was also employed in conducting Mantel-Haenszel test to adjust for possible confounding effect of age on the variables. Stratified analysis was performed on each professional group after which summary chi square and p value were computed. Level of statistical significance was set at P < 0.05.

## Results

### Sociodemographic characteristics of study participants

Table 1 shows socio-demographic characteristics of study population. The two hundred and seven female healthcare professionals who participated in this study were 141 nurses (68.1%), 45 doctors (21.7%), 13 laboratory scientists (6.3%), 4 pharmacists (2%) and 4 physiotherapists (2%). Age range of 182 participants (88%) who volunteered information on age was from 18 to 57 years (Mean = 37.5 ± 8.4). All participants possessed basic professional certificate while a few (10%) had additional post-graduate qualification. Ninety six percent of respondents were married.

**Table 1:** Sociodemographic characteristics of study participants

Variable	Doctors (number)	Nurses (number)	Others* (number)	Percentage
1. Profession	45	141	21	100
2. Age distribution (n = 182)				
a. < 20	0	2	0	1
b. 20–29	26	8	10	24
c. 30–39	13	67	8	49
d. 40–49	1	31	1	8
c. ≥ 50	0	14	1	8
3. Marital status				
a. Married	41	138	19	96
b. single	4	3	2	4
4. Professional qualification				
a. Basic	43	139	15	95
b. Postgraduate	2	2	6	5

Others* = Pharmacists, Laboratory scientists and Physiotherapists.

### Knowledge of risk factors and awareness of breast cancer

Tables 2&3 show participants responses to questions on breast cancer risk factors. Twenty-nine study participants (14%) had excellent knowledge of risk factors, 3% possessed very good knowledge, 45% had good knowledge while theremaining 37% had poor knowledge of risk factors assessed. Fifty-six percent among doctors had excellent knowledge and only 13% were considered as having poor knowledge. In contrast, 43% among nurses possessed poor knowledge and only 2(1%) had excellent knowledge. Doctors were the only group with satisfactory mean knowledge score of 74%. Mean knowledge score for nurses was 35% while other allied professionals scored 31%. One hundred and eighty-two participants (89%) identified increasing age as a risk factor while 169 (82%) recognised current use of oral contraceptive pills as breast cancer risk factor. Other risk factors were recognised by less than three-quarter of participants. Least recognized risk factors were Nulliparity and advanced age at first childbirth. Only 29% of participants were able to identify both as risk factors. Difference in knowledge score between doctors and nurses was statistically significant (p < 0.001). There was no statistically significant difference in knowledge score among nurses and other allied healthcare providers in this study (p = 0.6). Participant's profession was found to be associated with level of knowledge of breast cancer risk factors (Chi square = 78.48, p < 0.001) while age of respondents in this study was not (Chi square = 0.12, p = 0.72). Two hundred and five participants (99%) were aware of BSE. However, a lesser proportion (85%) was familiar with CBE. One hundred and eighty-eight participants (91%) were aware of mammography as a screening method for breast cancer. Level of awareness was however higher among doctors and Nurses (92.5%) as against that of the remaining participants (76%). Nearly all participants (91%) agreed that all females above the age of 40 years need to have regular breast cancer screening. Majority of participants believed that breast cancer is a major health problem in Nigeria. The question on the level of breast cancer awareness among the populace elicited varied responses. One-third among nurses in this study was satisfied with the level of awareness about breast cancer in the populace whereas only 9% among doctors and 19% among allied healthcare professionals were satisfied with current level of awareness. The difference in the responses of doctors and nurses to this question was statistically significant (P = 0.025).

**Table 2:** Participants correct knowledge of risk factors and awareness of breast cancer

Variable	Doctors(n = 45) Number(%)	Nurses(n = 141) Number(%)	Others*(n = 21) Number(%)	p-value
Risk factors				
Risk factors Increasing age	43(95.6)	121(85.8)	18(85.7)	0.091
Positive family history of breast cancer	39(86.7)	82(58.2)	12(57.1)	0.002
First childbirth at the age of ≥ 30 years	26(57.8)	19(13.5)	3(14.3)	< 0.001
Nulliparity	33(73.3)	18(12.8)	3(14.3)	< 0.001
Early menarche at the age of ≤ 12 years	33(73.3)	6(4.3)	2(9.5)	< 0.001
Late menopause at the age of ≥ 55 years	31(68.8)	8(5.7)	3(14.3)	< 0.001
History of previous benign breast lump	34(90.4)	92(65.2)	10(47.6)	0.115
Current use of oral contraceptive pills	36(80)	119(84.4)	14(66.7)	0.224
				
Awareness				
Aware of BSE	45(100)	139(98.6)	21(100)	0.623
Aware of CBE	38(84.4)	127(90.1)	17(81)	0.352
Aware of mammography	42(93.3)	130(92.2)	16(76.2)	0.48
Routine breast cancer screening is necessary for women > 40 years	43(95.6)	128(90.8)	18(85.5)	0.571
Breast cancer is a major health problem in Nigeria	39(86.7)	126(89.4)	16(76.2)	0.35
There is sufficient breast cancer awareness in Nigeria	4(8.9)	37(33.3)	4(19)	< 0.001

Others* = Pharmacists, Laboratory scientists and Physiotherapists.

**Table 3:** Correct knowledge of risk factors and awareness of breast cancer among nurses and doctors

Variable	Doctors(n = 45) Number(%)	Nurses(n = 141) Number(%)	p-value
Risk factors			
Increasing age	43(95.6)	121(85.8)	0.151
Positive family history of breast cancer	39(86.7)	82(58.2)	< 0.001
First childbirth at the age of ≥ 30 years	26(57.8)	19(13.5)	< 0.001
Nulliparity	33(73.3)	18(12.8)	< 0.001
Early menarche at the age of ≤ 12 years	33(73.3)	6(4.3)	< 0.001
Late menopause at the age of ≥ 55 years	31(68.8)	8(5.7)	< 0.001
History of previous benign breast lump	34(90.4)	92(65.2)	0.364
Current use of oral contraceptive pills	36(80)	119(84.4)	0.527
			
Awareness			
Aware of BSE	45(100)	139(98.6)	0.574
Aware of CBE	38(84.4)	127(90.1)	0.440
Aware of mammography	42(93.3)	130(92.2)	0.158
Routine breast cancer screening is necessary for women > 40 years	43(95.6)	128(90.8)	0.364
Breast cancer is a major health problem in Nigeria	39(86.7)	126(89.4)	0.406
There is sufficient breast cancer awareness in Nigeria	4(8.9)	37(33.3)	0.025

### Beliefs about breast cancer treatment

Responses of participants to questions on beliefs about breast cancer treatment are shown in tables 4&5. More than 80% of participants believed that breast cancer can be cured if detected early. Two-third held the belief that surgery is the most effective method of treatment for breast cancer. Among doctors, half believed that herbal or alternative medical therapy cannot cure breast cancer while a larger proportion (65%) among the remaining participants held similar belief. Forty two percent among doctors and 53.5% among the rest of participants believed that breast cancer can disappear following prayer. Only 54 (26%) participants were convinced that prayer cannot lead to disappearance of breast cancer. There was no statistically significant difference in response of doctors and nurses to question on whether surgery is the most effective method of treatment for breast cancer or not (p = 0.494). Similarly, difference in response of nurses and doctors to question on whether breast cancer can disappear following prayer or not was not statistically significant(p = 0.096).

**Table 4:** Participants positive beliefs about breast cancer treatment

Variable	Doctors(n = 45) Number(%)	Nurses(n = 141) Number(%)	Others(n = 121)	p-value
Breast cancer is curable if detected early	41(91.1)	121(85.5)	17(80.9)	0.053
Surgery is the most effective method of treatment for breast cancer	35(77.8)	108(76.6)	17(80.9)	0.806
Alternative or herbal medicine cannot cure breast cancer	22(48.9)	97(68.8)	13(61.9)	0.05
Breast cancer cannot disappear following prayer	10(22.2)	37(26.2)	7(33.3)	0.197

Others* = Pharmacists, Laboratory scientists and Physiotherapists.

**Table 5:** Positive beliefs about breast cancer treatment among Nurses and doctors

Variable	Doctors(n = 45) Number(%)	Nurses(n = 141) Number(%)	p-value
Breast cancer is curable if detected early	41(91.1)	121(85.5)	0.026
Surgery is the most effective method of treatment for breast cancer	35(77.8)	108(76.6)	0.494
Alternative or herbal medicine cannot cure breast cancer	22(48.9)	97(68.8)	0.008
Breast cancer cannot disappear following prayer	10(22.2)	37(26.2)	0.096

### Breast cancer screening practices

Tables 6&7 summarize breast cancer screening practices among respondents. Eighty three percent of participants in this study reportedly practice BSE at least once every month. Proportion of doctors and nurses who carry out regular BSE was higher than that of other participants. There was no statistically significant difference in BSE rate between doctors and nurses (p = 0.544). Frequency of CBE among participants was rather low with less than one-third affirming that they had CBE within the past one year. Majority of those that had CBE did so because of breast symptoms. Only 17 participants (8%) ever had mammogram before this study. Rate of reported BSE practice by participants in this study was not influenced by age (Chi square = 13.34, p = 0.2), knowledge of breast cancer risk factors (Chi square = 3.29, p = 0.19), profession (Chi square = 0.8, p = 0.93) or beliefs about breast cancer treatment (P > 0.05).

**Table 6:** Participants practice of screening methods

Variable	Doctors(n = 45) Number(%)	Nurses(n = 141) Number(%)	Others(n = 21) Number(%)	p-value
Performs BSE at least once a month	42(93.3)	135(95.8)	15(71.4)	< 0.001
Performed CBE within last 1 year	14(31.1)	35(24.8)	4(19)	0.539
Had mammography in the past	3(6.7)	13(9.2)	1(4.8)	0.717

Others* = Pharmacists, Laboratory scientists and Physiotherapists.

**Table 7:** Practice of screening methods among nurses and doctors

Variable	Doctors(n = 45) Number(%)	Nurses(n = 141) Number(%)	p-value
Perform BSE at least once a month	42(93.3)	135(95.8)	0.544
Performed CBE within last 1 year	14(31.1)	35(24.8)	0.765
Had mammography in the past	3(6.7)	13(9.2)	0.183

## Discussion

Excluding doctors, knowledge of breast cancer risk factors among female healthcare professionals in this study is poor. Reports from similar studies among female healthcare providers support this finding [6,16]. An earlier study in Lagos, Nigeria, found unsatisfactory level of knowledge about breast cancer risk factors among nurses [19]. In United Kingdom, a study of General Practitioners knowledge of breast cancer risk factors showed that more than half of the participants were able to correctly identify risk factors assessed [15]. Report from Australia, however, revealed that General Practitioners have limited knowledge of some aspects of breast cancer risk factors with only 25% of those interviewed in the study recognising increasing age as breast cancer risk factor [17].

Level of knowledge of breast cancer risk factors is similar among nurses, pharmacists, physiotherapists and laboratory scientists in this study. Nurses play key role in breast cancer information dissemination and care because of their more frequent interaction with patients and their relatives. Furthermore, this role has been expanded in some advanced countries with the evolution of specialist breast care nurse [21]. Although, they are generally better informed about breast cancer than non-healthcare providers, a report from Jordan found no significant difference in level of knowledge of risk factors between nurses and schoolteachers [22]. In the United States of America, Powe et al. [23] reported that significant number of myths and misperceptions related to breast cancer were prevalent within nursing and non-nursing college students. In addition, few nursing students reported obtaining information on common perception about breast cancer from their course work.

Participants in this study have positive perception of breast cancer treatment. Majority held the belief that early stages of the disease are curable and that surgery is the most effective method of treatment. These findings support the report from an earlier study among nurses in Lagos, Nigeria where 89% would agreed to have mastectomy should they develop breast cancer [19]. In a more recent study of women from a semi-urban neighbourhood in southern Nigeria, Okobia et al. [5] reported that 41% of participants believed that breast cancer is curable if detected early. In contrast, a study conducted among literate Nigerians almost a decade ago revealed that only 5% believed that cancer is curable when detected early [24].

Belief in the efficacy of traditional methods of therapy and prayer in the cure of breast cancer is widespread among females in developing countries and African-American women [7,12,25]. In this study, half of participants believed that prayer can lead to disappearance of cancer from affected breasts. Furthermore, only two-third is convinced that herbal or traditional mode of treatment cannot cure breast cancer. A study conducted among nurses in Lagos, Nigeria, found that more than two-third believed in prayer houses as places of effective treatment of breast cancer [19]. Wrong cultural and religious beliefs about breast cancer are significant factors in late presentation of the disease [7,12,25]. In a study by Mitchell et al. [12], strong religious beliefs were found to be common among women in Eastern North Carolina in United States of America. The report showed that a majority believed that God works through doctors to cure breast cancer. In addition, minority, who were mainly African-Americans, believed that medical treatment was unnecessary because only God could cure breast cancer. A significant proportion of female healthcare professionals in this study believed in efficacy of prayer and traditional or herbal therapy. This calls for concern because such beliefs could have negative effect on their role in creating appropriate awareness about breast cancer in Nigeria, a country where majority of those with the disease present with advanced stages [9].

Practice of BSE by participants in this study is commendable. Ninety-five percent carried out the procedure at least once every month. This is an improvement over 89% reported in an earlier study among nurses in Lagos [19]. However, lower rates of BSE were found among health workers who participated in a community based study in Port Harcourt, Southern Nigeria [20] and only 6% among healthcare workers in Iran perform BSE monthly [6]. Community-based studies in developing nations often report low rates of regular BSE among women [5,26]. In contrast, higher rates are commonly found among women from developed countries where breast cancer awareness is believed to be better [27,28].

Practice of CBE by study participants is rather poor. Only 26% have had the procedure in the past 1 year. Low rates of CBE were reported in earlier studies among nurses and community-dwelling women in Nigeria [5,19]. Azaiza et al [29] reported that barriers to regular performance of CBE by women include feeling of discomfort and embarrassment, belief that CBE is a painful procedure and belief that there is no cure for cancer.

Rates of BSE and CBE by participants in this study were found not to be influenced by age, profession or knowledge of risk factors. Although, association between older age and more frequent practice of BSE was reported by Jacob et al [27], other studies did not find such a relationship [5,26]. Reports also showed that adequate knowledge about breast cancer influences BSE practice positively [5,26,30].

Majority of participants in this study were aware of mammography as a screening method for early breast cancer. However, only 8% among participants ever had mammogram. Reports from developing nations often indicate low rates of mammography screening practice [5,26]. Okobia et al. [5] reported that none of the participants in a study among semi-urban community-dwelling women in Nigeria ever had mammography screening. In contrast, significantly higher rate of mammography screening is often reported among women in advanced countries [28,31].

Breast self-examination, CBE and mammography are recognised methods of screening for breast cancer and adoption of mammography screening has led to reduction in mortality from the disease in women over 50 years [10]. Although, some countries with population-based breast cancer screening program use all the three methods, others recommend use of either one or two [32]. The American cancer society guidelines for cancer screening recommend annual mammogram and CBE for women above the age of 40 years. Furthermore, monthly BSE is made optional with emphasis on the importance of breast self-awareness outside a structured BSE [11]. Although, regular BSE does not influence mortality from breast cancer [33], it assists women in detecting benign breast lumps and in creating more awareness about breast changes.

Women with breast cancer in Nigeria are relatively younger than their Caucasian counterparts [2,9]. Therefore, adopting mammography screening guidelines designed for Caucasian population may not be beneficial in Nigeria since a large proportion of women with breast cancer are younger than the recommended age group for screening. In many developing countries like Nigeria, priority is given to child and maternal health as well as control of communicable diseases in the allocation of funds. Considering the poor economy of these countries, provision of facilities for routine mammography screening of women at risk may not be justifiable. Adoption of routine and regular BSE and CBE appear to be more realistic and affordable methods of breast cancer screening in Nigeria.

Small proportion of study participants delayed return of the questionnaires for several hours. This may raise concern about reliability of their responses. However, efforts were made to ensure that majority responded promptly and returned the questionnaires immediately. The sample size in this study is skewed in favour of nurses and doctors because of relatively smaller number of other female healthcare professionals in the Institution. This challenge is addressed by comparing the two dominant groups separately in data analysis.

## Conclusion

Knowledge of breast cancer risk factors is satisfactory among doctors but inadequate among a large percentage of nurses and other allied healthcare professionals in this study. Considering their leading role in breast cancer awareness and information dissemination, efforts should be made by Government and Non-Governmental agencies to improve breast cancer knowledge among healthcare providers other than doctors. Frequent continuing medical education programmes on breast cancer at institutional level is desirable. In addition, greater emphasis needs to be placed on breast cancer in the curricula of nursing and other healthcare training institutions so that graduates of such schools are better informed about the disease.

Significant proportion of participants in this study believed in the role of prayer and alternative medical therapy in the cure of cancer. This is a reflection of high level of spirituality and belief in tradition among Nigerians. Programmes to educate religious leaders and alternative medical practitioners about breast cancer should be encouraged. The advantage of early presentation and treatment of patients suffering from breast cancer should be emphasized during such interactions.

Although, practice of BSE among participants in this study is satisfactory, female doctors, nurses and other allied healthcare professionals should be encouraged to adopt regular CBE as well. This will positively influence their role in motivating other women in the society who look upon them for advice and guidance in adopting the practice of screening methods.

## Competing interests

The authors declare that they have no competing interests.

## Authors' contributions

NAI designed the study, acquired data, performed statistical analysis and drafted the manuscript. OOO revised and contributed to the final draft of the manuscript and statistical analysis of data. All authors read and approved the final manuscript.

## Pre-publication history

The pre-publication history for this paper can be accessed here:

http://www.biomedcentral.com/1471-2407/9/76/prepub
